# Calf arteriovenous malformation presenting as an iliac artery aneurysm: A case report

**DOI:** 10.1016/j.ijscr.2023.109082

**Published:** 2023-11-20

**Authors:** Javad Salami, Ammar Atieh, Yaser Ftouni

**Affiliations:** Vascular and Endovascular Surgery Department, Sina Hospital, Tehran University of Medical Science, Iran

**Keywords:** Iliac artery aneurysm, Chronic venous insufficiency, Arteriovenous malformations

## Abstract

**Introduction and importance:**

Arteriovenous malformations (AVMs) are abnormal connections between arteries and veins. Common signs of AVMs include a pulsating mass, pain, ulceration, bleeding, and tissue necrosis. This case report discusses a rare presentation of an iliac artery aneurysm in a patient with an extensive calf AVM.

**Case presentation:**

A 35-year-old male presented with a pulsatile mass in the abdomen, along with symptoms of chronic venous insufficiency in the lower limb. He had undergone multiple surgeries for varicose veins in the past. Initially, external iliac artery aneurysm was diagnosed. Further assessment revealed the presence of an AVM in the calf. After multiple unsuccessful endovascular interventions, amputation was recommended. However, the patient opted for conservative management.

**Clinical discussion:**

AVMs are vascular malformations that are present from birth. Angiography is considered the gold standard for confirming the diagnosis of AVMs. As there is no consensus on the best treatment for AVMs, a multidisciplinary approach is recommended on a case-by-case basis. Delaying treatment can lead to serious complications and increase morbidity and mortality. Treating extensive AVMs that involve the entire extremity can be particularly challenging and often result in poor outcomes.

**Conclusion:**

The presence of varicose veins at a young age may indicate an underlying AVM. AVM can manifest in various ways, including arterial aneurysms. In severe cases, extensive AVMs may require limb amputation when other treatments fail.

## Highlights

• Arteriovenous malformations (AVMs) are vascular malformations present from birth.
• AVMs can be discovered incidentally or can lead to severe complications that may pose a threat to life or limb.
• Extensive calf AVM may present as an iliac arterial aneurysm.
• There is currently no consensus regarding the optimal treatment for arteriovenous malformations.
• Limb amputation may be necessary for extensive AVMs when other treatments fail to improve their symptoms.

## 1 Case presentation

**Fig. 1:**
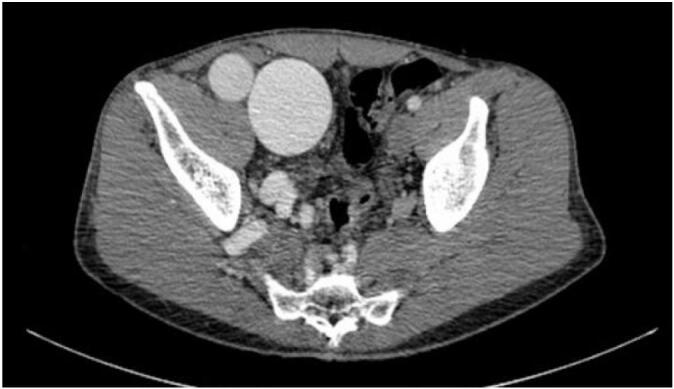
Aneurysm of iliac artery that caused the patients symptoms who presented with a pulsatile groin mass, along with severe dilation of right external iliac vein due to high flow AVM.

**Fig. 2:**
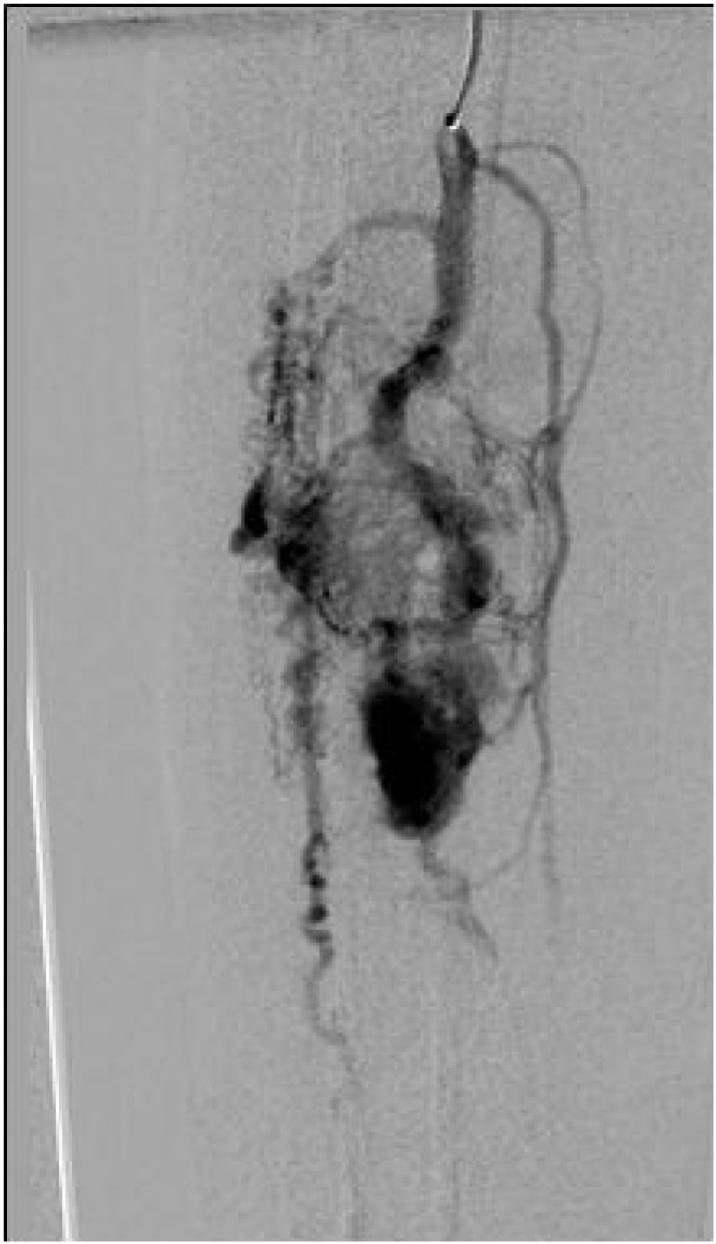
Late arterial phase demonstrates large AVM with opacification of the dilated peroneal artery and multiple dilated small vessels as well as early opacification of the aneurysmal peroneal vein.

**Fig. 3:**
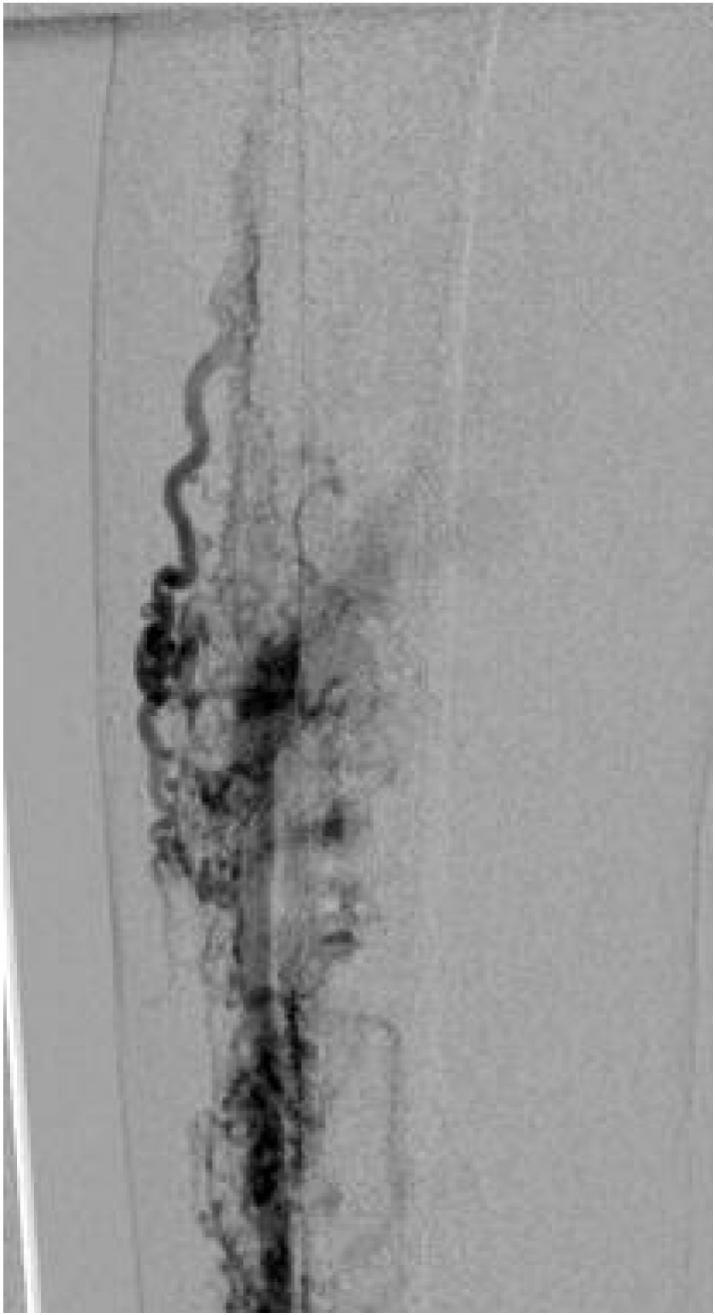
Selective angiography of posterior tibialis artery and AVM.

**Fig. 4:**
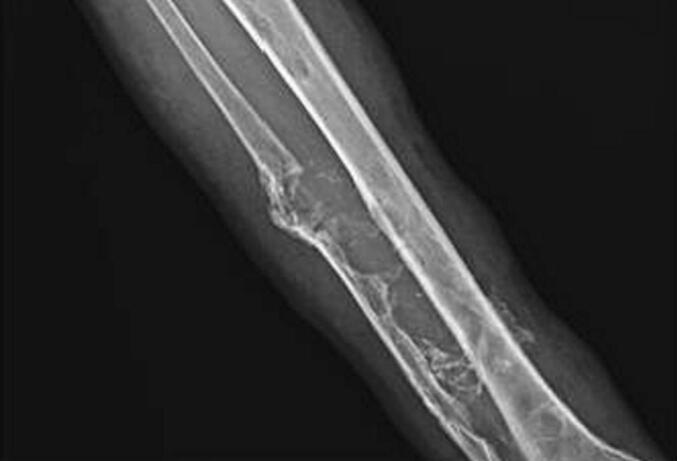
Erosion of fibula bone with adjacent calcification within AVM.

A 35-year-old male presented with a pulsatile mass in the right lower quadrant of the abdomen that had been present for several years and extended to the groin. He reported having undergone multiple surgeries for varicose veins at the ages of 9 and 24, but the details of these surgeries were unknown. The physical examination revealed a pulsatile swelling in the right lower quadrant of the abdomen. In the right lower extremity, a pulsatile mass was palpated in the popliteal fossa, and multiple small scars located below the knee, likely related to previous phlebectomy were noticed, in addition to symptoms consistent with chronic venous insufficiency, including leg swelling, brown-colored skin, ulcers, and varicose veins. The patient had previously undergone abdominal CT angiography elsewhere that showed external iliac artery aneurysm measuring 13 ∗ 5 cm along with dilated CFV and iliac veins (Fig. 1). Due to the presence of symptoms of chronic venous insufficiency, dilated iliac and femoral veins, and an unclear history of multiple surgeries for varicose veins, further assessment was conducted for the right lower limb with Doppler ultrasound. Based on measurements of diameter, flow volume, peak systolic velocities, and resistive indexes, the findings revealed the presence of an extensive calf arteriovenous malformation (AVMs) and a popliteal aneurysm. Selective angiography was performed to assist in selecting the appropriate approach and identifying the type of AVM. Due to the extensive AVM, popliteal artery aneurysm, multiple connections between supplying peroneal and tibialis arteries, severely dilated draining veins (Fig. 1, Fig. 2, Fig. 3), erosion of the adjacent fibula bone (Fig. 4), cardiomegaly reported in echocardiogram, and after the failure of multiple endovascular interventions and receiving a multidisciplinary opinion, amputation was recommended. However, the patient chose conservative management with compression stockings.

## 2 Introduction

Arteriovenous malformations are vascular malformations present from birth, characterized by abnormal connections between arteries and veins without capillaries in between. This results in a high-flow shunting of arterial blood flow into the draining veins. They can be discovered incidentally or can lead to severe complications that may pose a threat to life or limb. Some syndromes and mutations have been associated with AVMs [1]. Common clinical signs of AVMs in the trunk and extremities include a pulsating mass, pain, ulceration, bleeding, tissue necrosis, enlargement of draining veins, and venous hypertension and cardiac failure [2]. In this reported case, an iliac artery aneurysm presents as a complication of an extensive calf AVM.

## 3 Clinical discussion

Arteriovenous malformations are complex vascular anomalies that present a challenge in treatment due to their complex wide connections, proximity to vital structures, infiltration into deep and superficial tissues, and high rates of recurrence and treatment failure. Wide range of presentations has been reported, including a case of multiple serial aneurysms of the brachial artery with multiple arteriovenous malformations of the forearm [3].

**Fig. 5:**
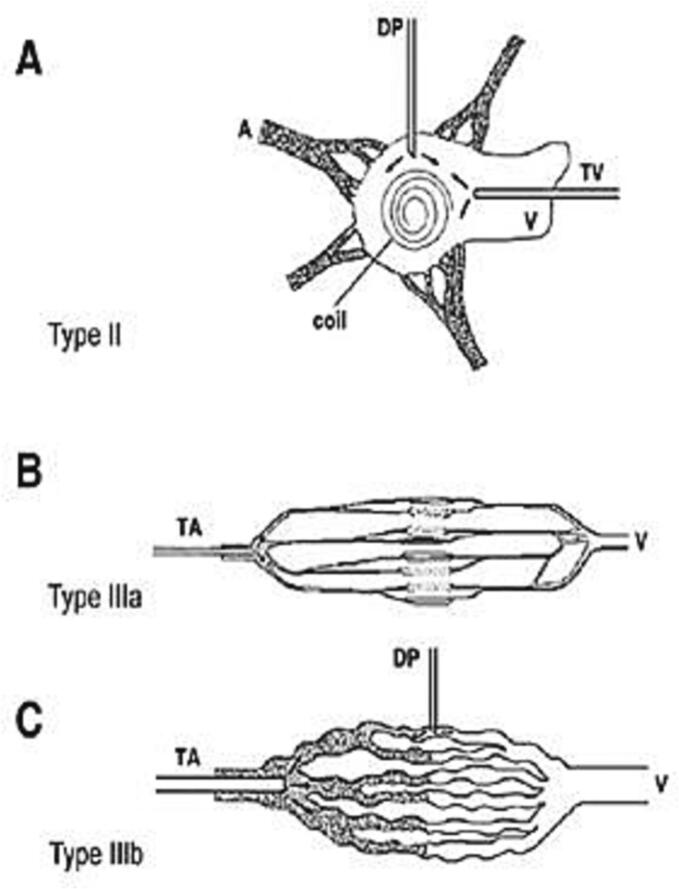
(A) Type II therapeutic approaches are transvenous (TV) and direct puncture (DP) (B) Only the transarterial (TA) approach is available for type IIIa AVMs because the fistula is too fine for direct puncture. (C) Type IIIb AVMs can be treated properly via transarterial (TA) and direct puncture (DP) approaches [5].

Early diagnosis is crucial to prevent irreversible destruction of adjacent tissues [1]. Angiography is considered the gold standard for confirming the diagnosis of AVM, as it can identify the feeding vessels, nidus, and early draining veins [2]. Houdart and colleagues [4] developed an angiographic classification for intracranial arteriovenous lesions, which was suitable for peripheral AVMs. According to this classification, AVMs were divided into three groups based on the number of feeding arteries and veins, as well as the form of vascular connections (plexiform arterial structure). Cho et al. [5] later modified this angiographic classification specifically for peripheral AVMs (Fig. 5). The case we are presenting is consistent with Type IIIb, where multiple arterioles shunt blood to multiple draining veins through multiple enlarged fistulae.

Type IIIb AVMs can be treated using transarterial and direct puncture approaches with ethanol, n-butyl cyanoacrylate, and Onyx. However, success rates vary and complications such as skin necrosis, nerve injury, pulmonary hypertension, infections and limb amputation may occur. Each of these available treatments is effective for specific patients. For Type IIIb AVMs, transarterial approach is preferred, while the transvenous approach is contraindicated.

Due to the emergence of endovascular approach, the open approach is now less commonly adopted and is reserved for cases where AVMs are superficial and small in size. Early aggressive diagnostic evaluation and treatment is recommended if there are any serious concerns about the risk of the AVM, even in very young children, before it progresses to the point of no return [9]. Treatment of AVMs can be challenging and may require multiple embolization procedures to manage persistent and recurrent lesions [6,7]. In some cases, up to 50 procedures have been performed before limb amputation was necessary [8]. Extensive AVMs involving the entire extremity are difficult to treat and usually have unfavorable outcomes [2], amputation is considered a last-option treatment and it is typically performed when complications arise and endovascular treatment fails to control symptoms. Delaying this procedure can lead to potentially life-threatening disorders and increase morbidity and mortality. There is currently no consensus on the optimal treatment for these malformations, and a multidisciplinary approach on a case-by-case basis is warranted particularly before undertaking an amputation.

In this case, a large AVM with multiple connections extending from the proximal to the distal parts of the calf is observed (Fig. 2, Fig. 3). Additionally, there is erosion of the adjacent fibula bone (Fig. 4), along with dilated venous and arterial systems and aneurysm formation in the popliteal and iliac arteries in association with cardiomegaly. After discussing the potential complications of further endovascular interventions and delaying definite treatment, and after receiving a multidisciplinary opinion, limb amputation was recommended. However, the patient chose conservative management. Although it may sound aggressive treatment, limb amputation could be the only life-saving option when other alternatives fail to improve symptoms and quality of life for AVM patients.

## 4 Conclusion

It is important to remember that the presence of varicose veins at a young age may be caused by more complex factors, such as arteriovenous malformation, rather than just incompetent varicose veins. AVM can have diverse presentations, and arterial aneurysm should be considered as one of these presentations, particularly when it is associated with venous dilation and severe venous insufficiency symptoms. In cases were other treatments fail to improve symptoms, limb amputation may be necessary for extensive AVM patients.

## Consent

Informed consent was obtained according and in guidelines of the declaration of Helsinki. Written informed consent was obtained from the patient as well as the institute for publication and any accompanying images. A copy of the written consent is available for review by the Editor-in-Chief of this journal on request.

## Ethical approval

Ethics committee approval is often not required for case reports in our institution.

## Funding

None.

## Author contribution

All authors contributed evenly to the conceptualization, drafting, data analysis, writing and proofreading of the research.

## Guarantor

Atieh Ammar.

## Research registration number

NA.

## Conflict of interest statement

The authors declare no conflict of interest.

This article has been reported in line with SCARE criteria [10].
